# Oxidation of polyunsaturated fatty acids to produce lipid mediators

**DOI:** 10.1042/EBC20190082

**Published:** 2020-07-03

**Authors:** William W. Christie, John L. Harwood

**Affiliations:** 1 James Hutton Institute, Invergowrie, Dundee, Scotland DD2 5DA, U.K.; 2 School of Biosciences, Cardiff University, Cardiff CF10 3AX, Wales, U.K.

**Keywords:** fatty acid metabolism, lipid mediators, polyunsaturated fatty acids

## Abstract

The chemistry, biochemistry, pharmacology and molecular biology of oxylipins (defined as a family of oxygenated natural products that are formed from unsaturated fatty acids by pathways involving at least one step of dioxygen-dependent oxidation) are complex and occasionally contradictory subjects that continue to develop at an extraordinarily rapid rate. The term includes docosanoids (e.g. protectins, resolvins and maresins, or specialized pro-resolving mediators), eicosanoids and octadecanoids and plant oxylipins, which are derived from either the omega-6 (*n*-6) or the omega-3 (*n*-3) families of polyunsaturated fatty acids. For example, the term eicosanoid is used to embrace those biologically active lipid mediators that are derived from C_20_ fatty acids, and include prostaglandins, thromboxanes, leukotrienes, hydroxyeicosatetraenoic acids and related oxygenated derivatives. The key enzymes for the production of prostanoids are prostaglandin endoperoxide H synthases (cyclo-oxygenases), while lipoxygenases and oxidases of the cytochrome P450 family produce numerous other metabolites. In plants, the lipoxygenase pathway from C_18_ polyunsaturated fatty acids yields a variety of important products, especially the jasmonates, which have some comparable structural features and functions. Related oxylipins are produced by non-enzymic means (isoprostanes), while fatty acid esters of hydroxy fatty acids (FAHFA) are now being considered together with the oxylipins from a functional perspective. In all kingdoms of life, oxylipins usually act as lipid mediators through specific receptors, have short half-lives and have functions in innumerable biological contexts.

## Introduction

Since the pioneering work of George and Mildred Burr (reviewed in [1]) some 90 years ago, it has been known that certain fatty acids are essential in human (and most other animal) diets. There are two types of essential fatty acids (EFAs), which belong to the *n*-3 (omega-3) and *n*-6 (omega-6) polyunsaturated fatty acid (PUFA) families (i.e. the last double bond in these acids is either three or six carbons from the methyl end of the chain). In the early experiments, fat-free diets were shown to give rise to a variety of physiological symptoms that could be alleviated by feeding linoleic (LA) and α-linolenic (ALA) acids. The reason why these fatty acids are essential in the diet is that humans and animals in general do not have the necessary desaturases (Δ^12^ and Δ^15^) to form them from oleic acid, i.e. to insert a new double bond after one that is pre-existing [2]. They are produced almost entirely in photosynthetic organisms that evolve oxygen—such as cyanobacteria, algae, mosses and higher plants. While LA and ALA can be regarded as the key EFAs of the *n*-6 and *n*-3 families, respectively, there may be a need for longer chain PUFAs, produced from these precursors by sequential elongation and desaturation reactions, for specific functions. Such acids (e.g. arachidonic (ARA), eicosapentaenoic (EPA) and docosahexaenoic (DHA) acids) are termed ‘conditionally essential’ [3]. Moreover, there has been much recent interest in providing dietary 20 or 22C PUFAs and also ensuring that the ratio of dietary *n*-6/*n*-3 PUFAs is maintained at 3-4, which is much lower than in many current ‘Western’ diets [4].

Polyunsaturated fatty acids are important components of phospholipids in membranes to which they impart desirable physical properties. However, a major reason why we need EFA and why they produce so many diverse effects is because they are metabolised to give rise to lipid signalling molecules [5]. In the main, the latter are 20C (eicosanoids) or 22C (docosanoids) oxygenated derivatives, collectively termed ‘oxylipins’. Although linoleic acid is an important constituent of skin lipids [6], where it has vital functions, much of the dietary need for EFAs is to make longer-chain lipid signalling molecules. Nevertheless, there are a number of important signalling molecules in animals, which are formed from linoleic acid or α-linolenic acid *per se* (e.g. octadecanoids) [7].

In this article, we briefly describe the conversion of *n*-3 and *n*-6 PUFAs into various classes of lipid mediators. We also summarise the latter’s biological actions. More details of the metabolism and function of these lipid mediators will be found in subsequent chapters that cover eicosanoids, isoprostanes, specialised pro-resolving mediators (SPMs), endocannabinoids and jasmonates.

## Conversion of essential fatty acids into eicosanoids

**Figure 1:**
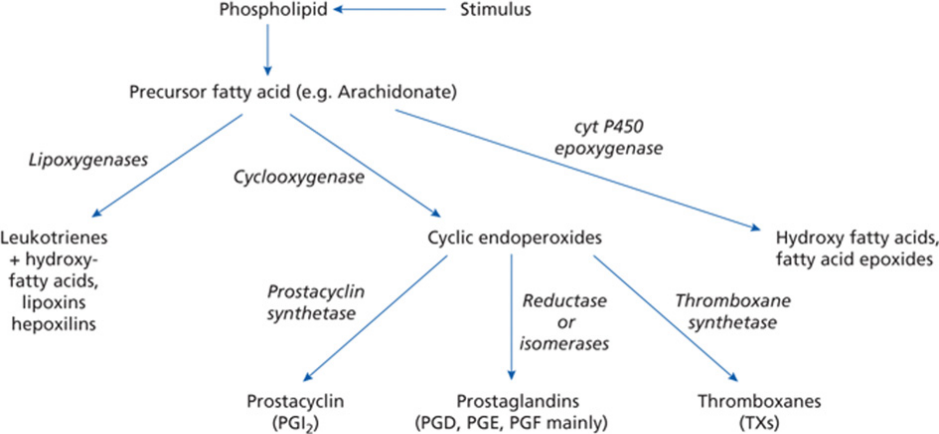
Generation of the classic eicosanoids (taken from [2] with permission)

A simplified picture of the generation of classic eicosanoids (derived from the Greek for 20) is shown in Figure 1. Three different types of oxidation reactions utilise a 20-carbon unesterified fatty acid precursor, such as arachidonic acid (ARA, the main *n*-6 precursor) or eicosapentaenoic acid (EPA, the main *n*-3 precursor). These involve lipoxygenase, cyclooxygenase and cytochrome P450 oxidase or epoxygenase enzymes [8].

Before reaction occurs, the PUFAs are usually released from membrane phospholipids mainly by phospholipase A_2_ (PLA_2_) action. A considerable number of PLA_2_ enzymes have been characterised and shown to occur in several unrelated protein families [9]. Overall, they can be broadly categorised into cytosolic calcium-dependent PLA_2_ (cPLA_2_), cytosolic calcium-independent PLA_2_ (iPLA_2_), and secreted PLA_2_ (sPLA_2_). The concentration of non-esterified PUFAs, such as arachidonic acid, in cells is normally far below the *K*m of enzymes such as cyclooxygenase (prostaglandin H_2_ synthase) so activation of hydrolytic enzymes, especially cPLA_2_, is a key regulatory reaction. For the 20C PUFAs, ARA and EPA, different lipid classes contain important relative concentrations. Thus, for ARA, two phosphoglyceride classes are important sources—phosphatidylcholine (the most abundant phosphoglyceride in most mammalian membranes) and phosphatidylinositol (and its phosphorylated derivatives) by virtue of the high concentration of ARA at the *sn*-2 position together with the specificity of cPLA_2_α [2]. Other potential sources of ARA or EPA are the plasmalogens but because these lipids are poor substrates for PLA_2_, they are usually hydrolysed by plasmalogenase (alkylglycerol monooxygenase) first [10,11].

Release of non-esterified PUFAs from membrane lipids can be enhanced by specific physiological stimulae (e.g. adrenaline, angiotensin II, certain antibody–antigen complexes) or non-specific pathological conditions. Of the many lipases that can be activated in this way, cPLA_2_α appears the most important as it has a marked specificity for phospholipids containing arachidonic acid in the *sn*-2 position. Hormonally induced mobilisation of Ca^2+^ leads to the movement of the enzyme from the cytosol to the endoplasmic reticulum and the nuclear envelope. Its activity is increased by phosphorylation. Two specific lipids—ceramide 1-phosphate and phosphatidylinositol 4,5-*bis*phosphate—bind to the enzyme and modify both its activity and its translocation within cells [12].

Secretory PLA_2_ (sPLA_2_) is also stimulated by Ca^2+^ but at the higher concentrations found outside the cell. The enzyme is rather non-specific towards different phosphoglycerides and towards the fatty acid present at the *sn*-2 position. There are suggestions that cPLA_2_ is involved in the rapid response in prostaglandin synthesis while sPLA_2_ is involved at later stages of prostaglandin stimulation after tissues have been activated further by cytokines, growth factors or inflammatory factors.

## Cyclooxygenase activity

**Figure 2:**
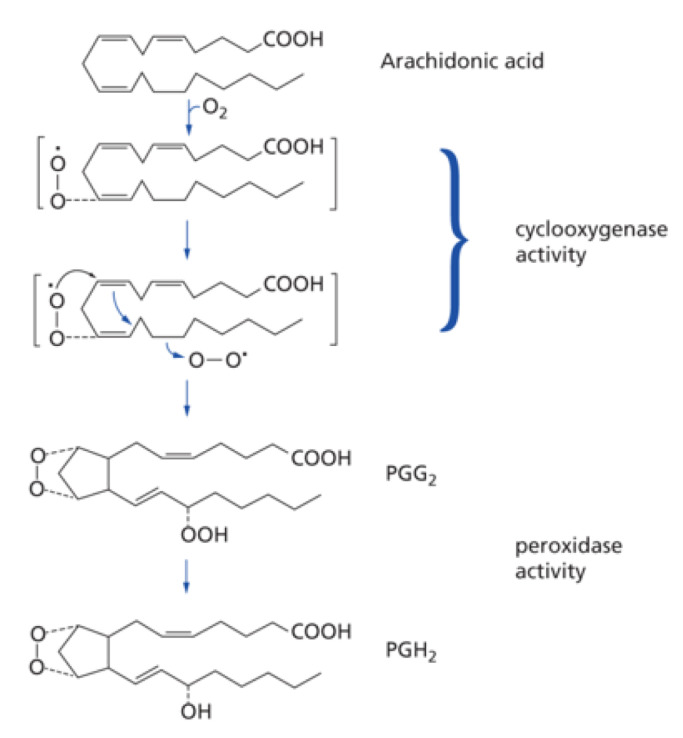
Cyclooxygenase activity (taken from [2] with permission)

Once the precursor fatty acid (ARA or EPA usually) has been released from membrane lipids, it can be oxidised by cyclooxygenases (more correctly termed prostaglandin endoperoxide H synthases) [8,13]. Two reactions are catalysed by a single enzyme—a cyclooxygenase reaction where two molecules of oxygen are added to the substrate and a second peroxidation (Figure 2). In the case of ARA, prostaglandin PGG_2_ is the intermediate and PGH_2_ the first product.

**Figure 3:**
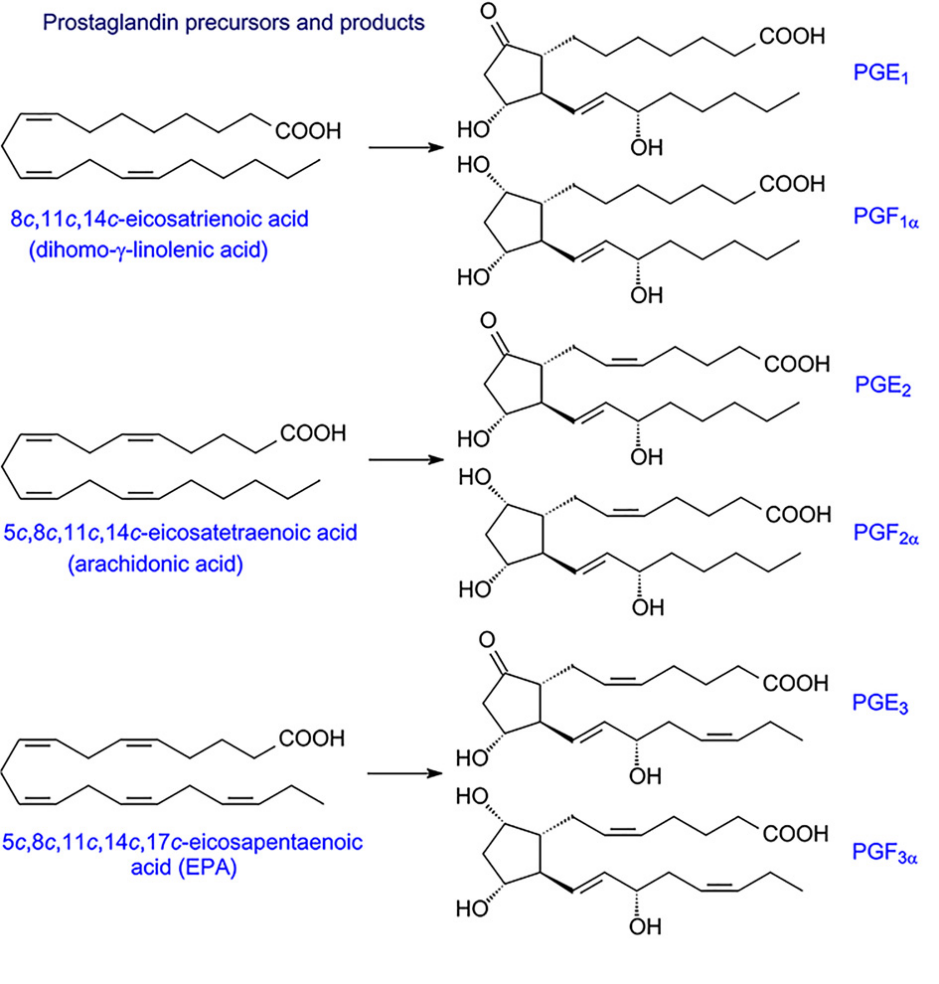
Prostaglandin precursors and products

**Figure 4:**
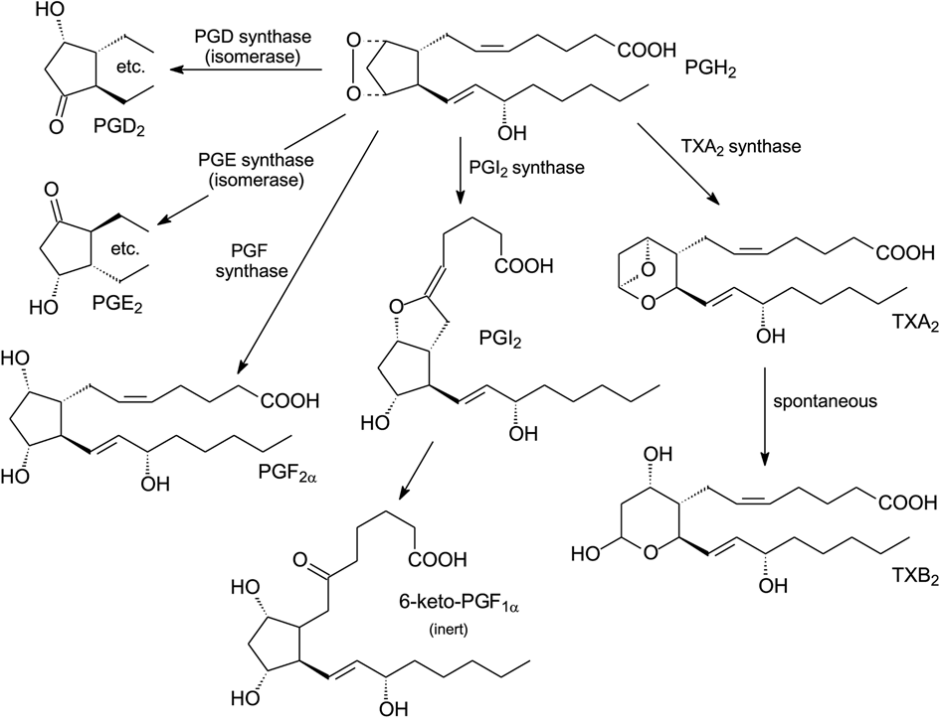
Conversion of cyclooxygenase endoperoxide products to different types of eicosanoids (adapted and re-drawn from [2])

There are two major human cyclooxygenase isoforms, COX-1 and COX-2, which are haemoproteins and act as homodimers of 576 and 581 amino acids, respectively. COX-1 is constitutively expressed in many mammalian tissues. It is thought to be responsible for the formation of prostaglandins involved in the general regulation of physiological events. COX-2, on the other hand, is present at low levels until induced by inflammatory stimuli such as cytokines, endotoxins, tumour promoters and some lipids. Both isoforms have similar *V*_max_ and *K*_m_ values for ARA, undergo suicide inactivation and their reactions can be initiated by hydroperoxide. The COXs will use a broad range of 18 or 20C PUFA substrates although their substrate selectivities are somewhat different. For example, COX-2 needs lower concentrations of hydroperoxide for activation and has a wider substrate specificity (including those relevant to endocannabinoid metabolism) than COX-1 [14]. Moreover, COX-2 has a greater capacity to oxidise a number of PUFAs that are poor substrates for COX-1 including various *n*-3 PUFAs [14–16]. When acetylated by aspirin, COX-2 in contrast with COX-1 can catalyse a lipoxygenase-type reaction involved, for example, in the formation of various SPMs (see later). Furthermore, the products of COX reactions will also relate to the balance of substrates available. Some examples of prostaglandin precursors and their products are shown in Figure 3. The endoperoxide products, in turn, can form a host of different products (see Figure 4).

## Cyclooxygenase endoperoxide products can be converted into various eicosanoids

Three general types of prostanoids can be synthesised from the endoperoxide produced by COX-1 or COX-2 (Figure 4). Prostaglandins PGD_2_, PDE_2_ and PGF_2_ are formed from PGH_2_, itself produced from ARA [17]. An alternative prostaglandin, PGI_2_ (also known as prostacyclin) [18,19] has a distinct function in promoting vasodilation and inhibiting platelet aggregation. Together with its action in inhibiting smooth muscle proliferation, PGI_2_ contributes to myocardial protection.

Instead of prostacyclin formation, PGH_2_ can be converted to thromboxane A_2_ via TxA_2_ synthase. TxA_2_ is extremely labile and rearranges spontaneously with a half-life of approximately 30 s to a stable but physiologically inert TxB_2_ (Figure 4). Because TxA_2_ causes platelet aggregation [20], there have been considerable efforts in searching for inhibitors of its synthesis. Since TxA_2_ and PGI_2_ have antagonistic effects on thrombosis and atherogenesis, it is obvious that their balance is essential for good cardiovascular health and maintenance of vascular homeostasis [21]. Thus, TxA_2_ is synthesised mainly in platelets and its production is enhanced during platelet activation to promote aggregation and vasoconstriction. On the other hand, prostacyclin (PGI_2_) is the main prostanoid produced by vascular endothelial cells. It will inhibit platelet aggregation and contributes substantially to cardiovascular protection (see [2,22]).

## Prostanoids have receptors that mediate their actions

In the last two decades, several prostanoid receptors have been identified and partly characterised [23–25]. For prostaglandins, ten receptors have been characterised. In the case of PGE_2_, four receptors (EP1–4) have been identified and each has a different mechanism of action. Other receptors have been identified for PGD_2_, PGF_2α_, PGI_2_ and TxA_2_. The receptors have seven transmembrane segments and belong to the G protein-coupled receptor (GPCR) family which constitute the largest family of receptors in humans (approximately 800 coded in the human genome).

Apart from prostanoids [26], receptors have been identified for other lipid mediators [27]. For example, in the case of leukotrienes, LTB_4_ has BLT1 (high affinity) and BLT2 (low affinity and less specific) receptors while the cysteinyl leukotrienes have five (CysLT1, CysLT2, P2Y12, GPR17 and GPR99). Lipoxin [28] and SPMs [29] also have identified receptors.

By using knockout mice, precise functional roles for the individual receptors are being elucidated.

## Prostanoids are produced *in situ* and are rapidly catabolised

The prostanoids can be regarded as ‘local hormones’. They are produced within tissues and have their main actions in that locality. In general, prostanoids have very short half-lives *in vivo*. For example, PGE_2_ and PGF_2α_ are rapidly metabolised and do not survive a single pass through the circulation! The lung plays a major role in the catabolism with oxidation of the hydroxyl group at C15 being the usual target. This is followed by attack of the 13-double bond and then beta- and omega-oxidation. For the major active prostaglandins, their blood concentrations are less than 10^−10^M, emphasising their activity as local hormones (or autocoids). Moreover, unlike conventional hormones, they are produced in almost every cell in the body. The prostanoids are transported out of cells via carrier-mediated mechanisms and, once in the circulation, are deactivated rapidly [2,8].

## Aspirin and non-steroidal anti-inflammatory drugs

Aspirin (acetylsalicylic acid) was originally utilised as a substitute for salicylate medicines which had been used for their health properties for 3,500 years [30,31]. Since 1897, when Bayer produced it, aspirin has been one of the World’s most commonly used medicines (approximately 40,000 tonnes or up to 120 billion pills per year!).

Of the various non-steroidal anti-inflammatory drugs (NSAIDs) (e.g. aspirin, ibuprofen, indomethacin, diclofenac), aspirin is the best known. NSAIDs will compete with PUFA substrates for the reaction site of COX enzymes but, in general, the therapeutic anti-inflammatory action of NSAIDs is caused by inhibition of COX-2. In contrast, the simultaneous inhibition of COX-1 causes most of the unwanted side-effects, such as gastric ulceration [32]. Once bound to the COX-1 active site, aspirin will cause irreversible inactivation through acetylation of serine 530. For COX-2, the acetylation reaction still allows oxygenation of ARA, in a similar manner to that of a lipoxygenase, but prostaglandin PGH_2_ is not formed (see section on resolvins).

Unlike aspirin, most of the other NSAIDs cause reversible inhibition of COX and compete with substrates such as ARA. Because NSAIDs inhibit both COX enzymes and it would be much more useful to only inhibit COX-2, considerable effort has been devoted to finding selective inhibitors [32,33]. Some success was achieved initially [34], but such compounds caused unwanted cardiovascular effects [35], which led to the clinical withdrawal of the initial products.

## Lipoxygenase activity will produce hydroxy-eicosatetraenes, leukotrienes and other signalling molecules

Lipoxygenases can catalyse three different types of reactions (dioxygenation of lipids to give hydroperoxides; hydroperoxidation of the latter into keto lipids; formation of epoxy leukotrienes via leukotriene synthase reaction) due to their multifunctional nature. Several lipoxygenases (LOXs) occur in mammals and, in humans, six main family members have been listed—5-LOX, 12-LOX, 12/15-LOX (15-LOX type 1), 15-LOX type 2, 12*R*-LOX and epidermal LOX [36]. There are seven LOXs in mice. Orthologues of the same gene have different reaction selectivities in different species. This can often compromise extrapolation of data from animal experiments to human conditions. Each of the lipoxygenase proteins in animals has a single polypeptide chain of 75–80 kDa mass. The N-terminal ‘beta-barrel’ domain functions in substrate acquisition while a larger catalytic domain has a single non-heme iron atom which is bound to conserved histidine residues and to the carboxyl group of a conserved isoleucine at the C-terminus of the enzyme. The iron is active in the ferric state. For 5-LOX and 8-LOX activities the substrate ARA enters carboxyl group first while for 12-LOX or 15-LOX the methyl terminus enters the active site. While the PUFA substrate is held in a tight channel, smaller channels direct molecular oxygen towards the selected carbon to allow formation of specific hydroperoxy-eicosatetraenes (HPETEs), which are subsequently reduced to hydroxy-eicosatetraenes (HETEs). Each lipoxygenase acts with high regio- and stereo-specificity to produce HETE with distinctive biological functions in particular tissues.

**Figure 5:**
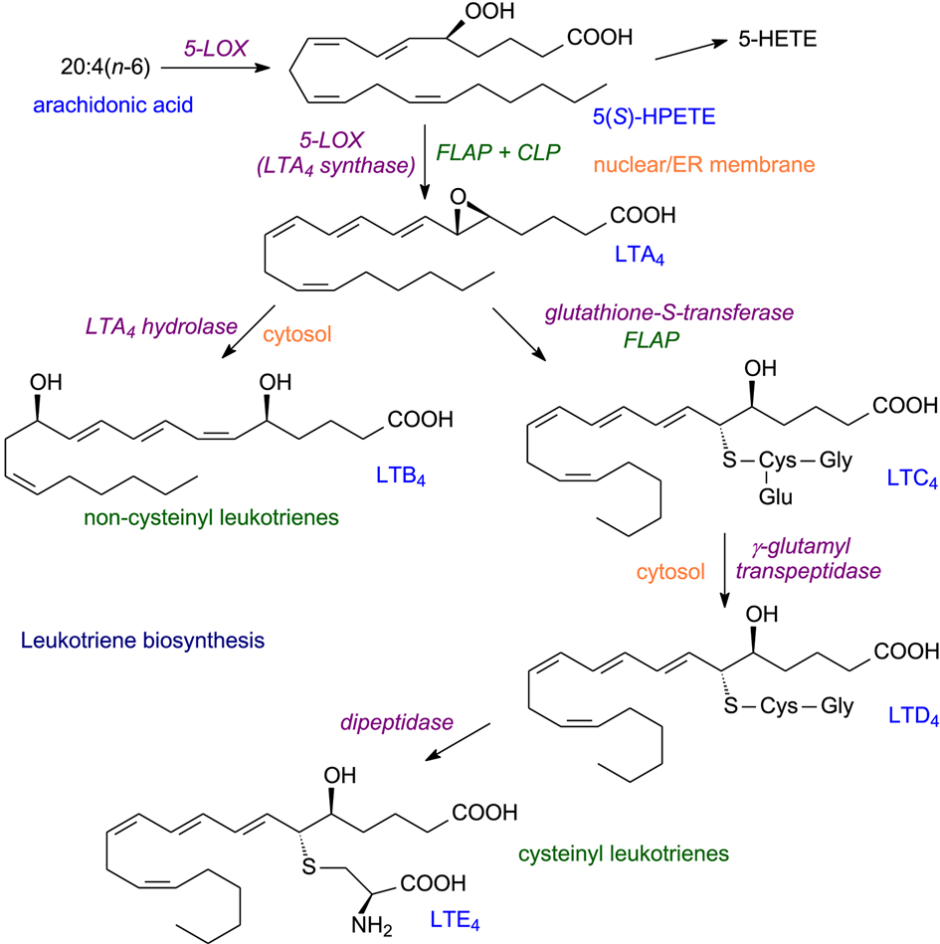
Leukotriene biosynthesis

For formation of leukotrienes, 5-LOX is the key enzyme [37,38]. As for prostanoids, a non-esterified (free) fatty acid is the substrate. A two-step concerted reaction begins leukotriene formation (Figure 5) [8]. The first step is a dioxygenation of C5 to create, in the case of ARA, 5*S*-hydroperoxy-6*t*,8*c*,11*c*,14*c*-eicosatetraenoic acid (5-HPETE). For the second step, two accessory proteins are needed. These are the 5-lipoxygenase activating protein (FLAP) and coactosin-like protein (CLP). With the aid of these two proteins, 5-LOX converts 5-HPETE into 5, 6-epoxy-7*t*,9*t*,11*c*,14*c*-eicosatetraenoic acid (5-HETE) or leukotriene A_4_ (LTA_4_) (Figure 5).

The unstable LTA_4_ appears to have little biological function on its own but can be metabolised in two ways to yield physiologically important leukotrienes. First, it can be hydrolysed by LTA_4_ hydrolase, a zinc metalloprotein, to form the dihydroxy acid LTB_4_ [39]. Secondly, LTA_4_ is converted to a ‘cysteinyl leukotriene’ by the addition of gamma-glutamyl-cysteinyl glycine and with the assistance of FLAP, this yields LTC_4_ which can be reacted with a transpeptidase to remove glutamate and yield LTD_4_. Finally, a dipeptidase forms the final ‘cysteinyl leukotriene’ LTE_4_.

The leukotrienes have a variety of important biological effects [40]. LTB_4_ is a potent chemotactic agent and is one of the first signals that attract innate immune cells such as leukocytes to the site of insult. In contrast, LTB_5_ (which is formed from EPA) strongly inhibits the pro-inflammatory actions of LTB_4_. Leukotriene LTC_4_, together with other ‘cysteinyl leukotrienes’ (LTD_4_, LTE_4_) are the slow-acting substances of anaphylaxis, originally reported nearly 100 years ago. These lipid mediators exert a range of pro-inflammatory actions including constriction of airways and vascular smooth muscles. LTD_4_ and LTE_4_ are overexpressed in several types of cancer and are considered tumorigenic.

**Figure 6:**
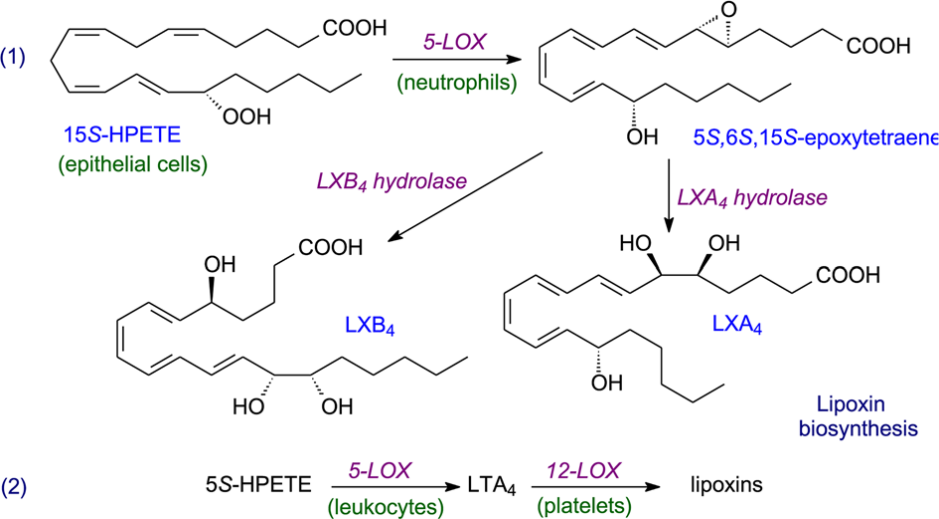
Examples of lipoxin biosynthesis

Instead of leukotrienes, ARA can form lipoxins, which are trihydroxy-eicosatetraenoic acids where the four double bonds are in conjugation (LXA_4_, LXB_4_) [41]. These are considered one of a group of specialised pro-resolving mediators (SPMs) which include resolvins, protectins and maresins (see later). Oxidation of ARA needs two different types of lipoxygenase in this case (see an example in Figure 6), and as few cell types have both of the necessary lipoxygenases, the synthesis of lipoxins needs trans-cellular pathways [42]. In such pathways, because a single cell lacks all of the enzymes necessary for a metabolic sequence, it has to combine with another cell type to complete a particular conversion. Of course, such cellular interactions need appropriate transport mechanism(s) associated with the pathway.

Two other minor classes of lipoxygenase products are the eoxins and the hepoxilins. Eoxins are related to the cysteinyl-leukotrienes and are products of the 12/15-LOX in human eosinophils and mast cells. They are potent pro-inflammatory agents [43]. Hepoxilins are especially important in human epidermis [44,45] and are made in one of two pathways involving 12-LOX. Compounds analogous to those formed from ARA can be produced from EPA or DHA and, in skin, linoleate is an important substrate [46].

## Cytochrome P450 oxidases, HETEs and epoxyeicosatetraenoic acids

**Figure 7:**
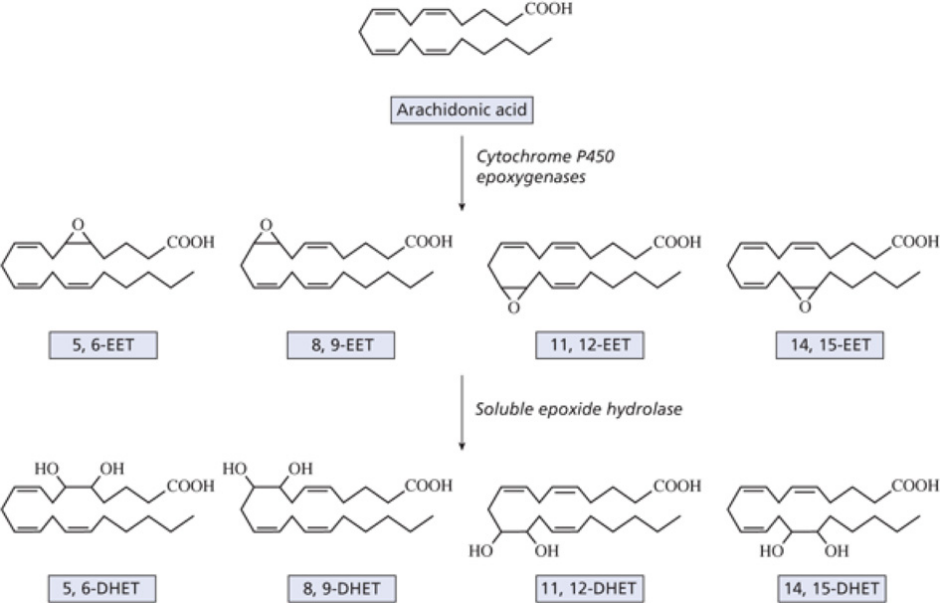
Metabolism of arachidonic acid by the cytochrome P450 epoxygenase pathway (taken from [2] with permission)

In addition to cyclooxygenase or lipoxygenase activity, a third oxygenation of relevant PUFAs involves cytochrome P450 oxidases (Figure 1) [8,47,48]. These enzymes are membrane-bound hemoproteins that transfer a single oxygen to the substrate carbon i.e. they are monooxygenases which produce water as a second product. NADPH is also needed and the transfer of electrons requires a NADPH-cytochrome P450 reductase. Various cytochrome P450 oxidases can produce a mixture of hydroxy-eicosatetraenes (HETEs) (Figure 7). The balance of HETEs generated depends on the tissue, cell type and the catalytic efficiency of the individual cytochrome P450 oxidase isoforms [49]. In addition to their function in generating HETE isomers, the cytochrome P450 oxidases have other important roles in lipid metabolism [50].

For ARA, three types of reaction can occur. First, a series of HETE products can be formed with *cis-trans* conjugated diols containing a hydroxyl group at one of six positions (5, 8, 9, 11, 12 or 15). Second, omega or omega-1 hydroxylases introduce a hydroxyl group at carbons 20 or 19, respectively, although minor activities with other oxidases can produce 16, 17 or 18 hydroxyl products. 20-HETE is pro-inflammatory and has largely detrimental functions, for example in increasing hypertension, in promoting systemic vasoconstriction and in tumour growth. It regulates vascular smooth muscle and endothelial cells by influencing their proliferation, migration, survival, and tube formation, acting via a specific G protein receptor (GPR75).

Third, cytochrome P450 oxidases can form a series of *cis*-epoxyeicosatrienoic acids (EETs) (14,15-, 11,12-, 8,9- and 5,6-EETs). Depending on the cytochrome 450 oxidase isoform, different EETs may predominate although most enzymes can produce all four isomers. The various EETs have major functions as autocrine and paracrine effectors in the cardiovascular and renal systems, which are believed to be largely beneficial. Because of the anti-hypertensive, fibrinolytic and anti-thrombotic properties of EETs, their presence in red blood cells has important implications for the control of circulation and the physical properties of the circulating blood. Both *cis*- and *trans*-EETs are synthesised and stored in erythrocytes, and they are produced and released in response to a low oxygen concentration as during exercise, for example.

EETs are rapidly metabolised *in vivo* to the corresponding dihydroxyeicosatrienoic acids (DHETs). This involves epoxide hydrolases, of which there are two isoforms, one membrane-bound and one soluble. The DHETs formed were once assumed to be inactivation products but now seem to have some biological activity of their own.

**Figure 8:**
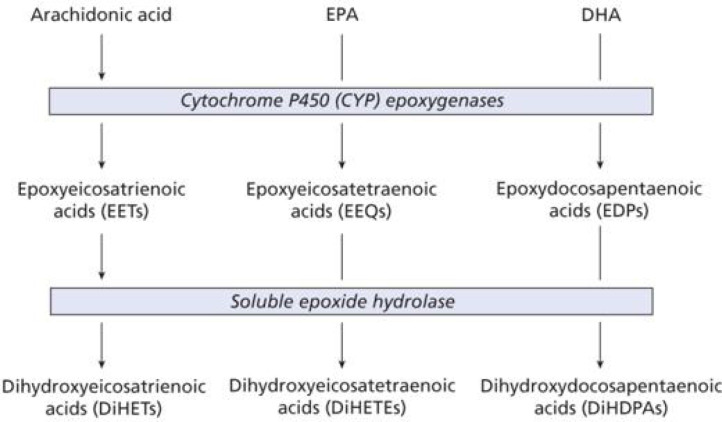
Action of CYP450 oxidases on EPA and DHA (taken from [2] with permission)

Historically, much of the original research with cytochrome P450 oxidases (as with cyclooxygenases and lipoxygenases) has focussed on ARA [48]. However, they have important activities with *n*-3 PUFAs such as EPA and DHA (Figure 8). In fact, the latter PUFAs are often the preferred substrates for some of the cytochrome P450 oxidases. Since their products often compete with metabolites from ARA, they can have beneficial effects, e.g. in alleviating pain. Such functions may account for some of the advantages of consuming significant dietary *n*-3 PUFAs. In addition, hydroxyoctadecadienoic acids (octadecanoids), which are formed by oxidation of linoleic acid, have a role in inflammation associated with important diseases, such as metabolic syndrome and cancer [7].

## Specialised pro-resolving mediators

**Figure 9:**
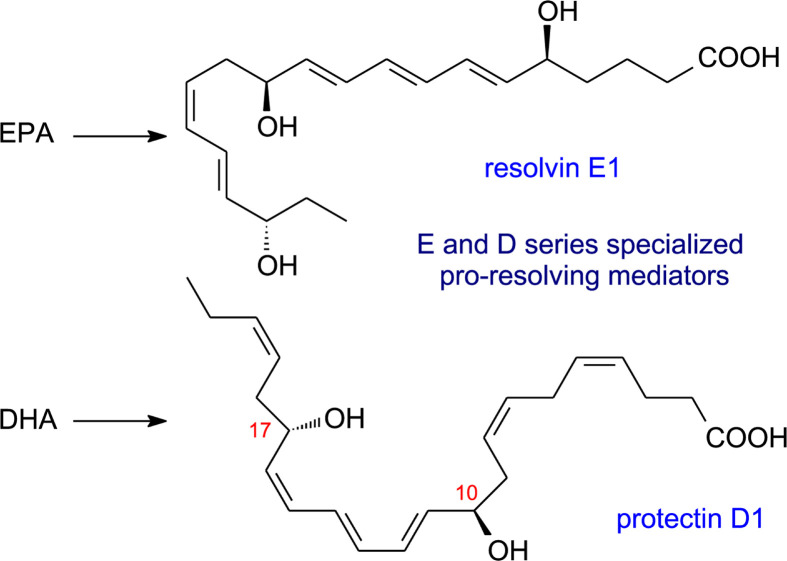
Examples of specialised pro-resolving mediators (SPMs)

**Figure 10:**
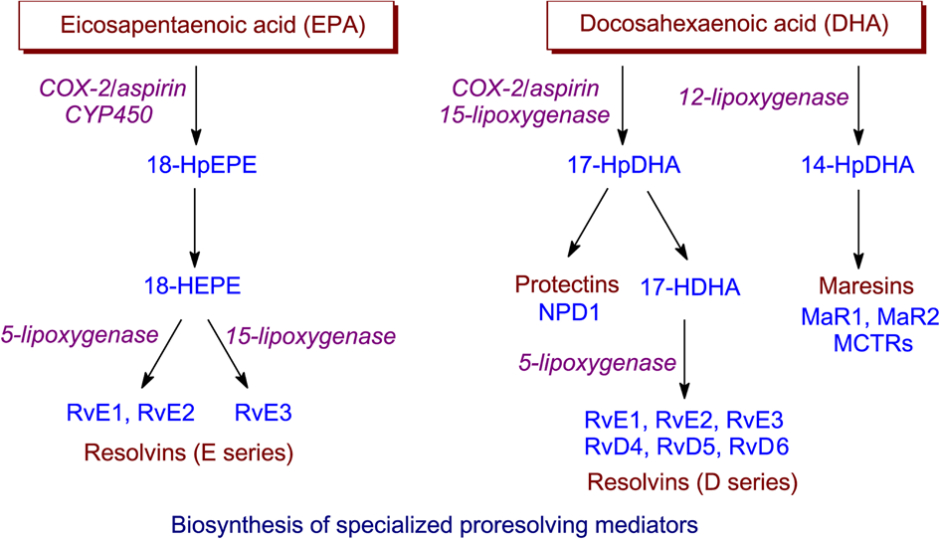
Biosynthesis of SPMs

Specialised pro-resolving mediators (SPMs), so-called because of their intimate involvement in the resolution stage of inflammation, have been mentioned already when discussing lipoxins in connection with lipoxygenase action. Over the last 15 years there has been increasing interest in the discovery and, subsequently, elucidation of the biosynthesis and biological function of other SPMs—protectins, resolvins and maresins [51]. In contrast with lipoxins, which are formed from the *n*-6 PUFA ARA, these other three groups of lipid mediators have very long chain *n*-3 PUFAs as their precursors, i.e. EPA, *n-*3 docosapentaenoic acid (DPA) and DHA. Oxylipins derived from EPA are designated SPMs of the E series while those from DHA are SPMs of the D series (Figure 9). An overall picture of the formation of protectins, resolvins and maresins by dioxygen-dependent oxidation is shown in Figure 10, and it will be seen that the same three types of oxidising enzymes discussed previously are all involved but with a preponderance of lipoxygenase reactions.

### Protectins

When studies were first made of metabolite formation from DHA in brain tissue following aspirin treatment, new docosanoids were discovered. These were dihydroxylated *E*,*E*,*Z*-docosatrienes and were termed ‘neuroprotectins’ because of their protective action and their formation in nervous tissue such as brain [52] where DHA is a major fatty acid [53,54]. Since then, similar docosanoids have been found in many tissues so ‘protectins’ is the preferred nomenclature [55,56].

**Figure 11:**
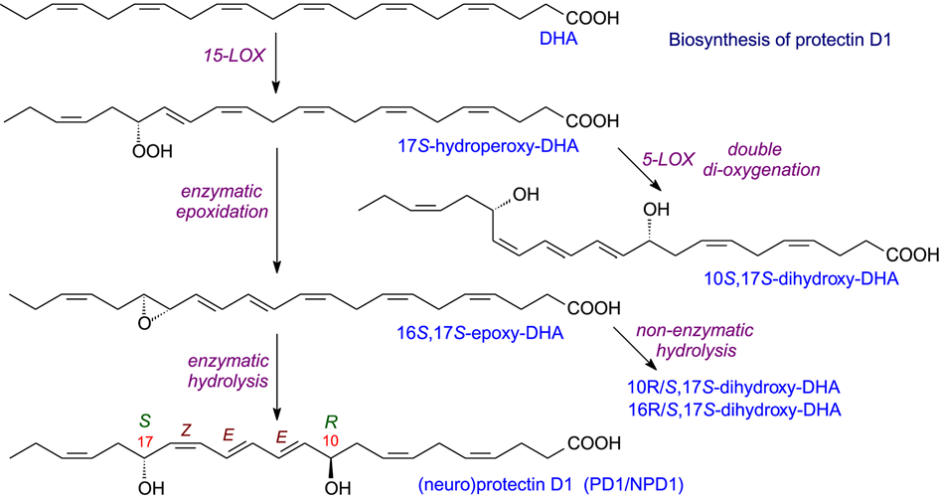
Biosynthesis of protectin D1

The biosynthetic pathway to protectin D1 (PD1, previously neuroprotection D1 or NPD1) is shown in Figure 11. Oxidation by 15-LOX yields a 17*S*-hydroperoxy-DHA that is converted into an epoxide and then hydrolysed. All the intermediates have very precise stereochemistry, which is also necessary for the biological activity of PD1. As illustrated in Figure 11, alternative reactions are possible via 5-LOX or non-enzymatic hydrolysis and these yield products which also have significant biological activity. Of interest is the reaction of 5-LOX which, when uncoupled from FLAP produces the SPM mediator (10*S*,17*S*-dihydroxy-DHA) rather than the inflammatory leukotriene B_4_. In addition to DHA, both 22:5(*n*-3) and 22:5(*n*-6) are good substrates for the 15-LOX. Their products as well as an omega-hydroxy analogue of PD1 (produced by CYP1) are all potent anti-inflammatory compounds.

### Aspirin-triggered protectins

Instead of the 17*S* intermediate produced by 15-LOX (Figure 11) in the production of PD1, aspirin treatment allows production of the 17*R* epimer (i.e. 10*R*,17*R*-dihydroxy-docosa-4*Z*,7*Z*,11*E*,13*E*,15*Z*,19*Z*-hexaenoic acid) from DHA. As mentioned before, aspirin is a classic inhibitor of COXs when it acetylates the active site. However, whereas it irreversibly inhibits COX-1, it only partially blocks the active site of COX-2, which retains lipoxygenase activity similar to that of 15-LOX but with the oxygen insertion in the *R*- rather than the *S*-configuration. With ARA as substrate the 15*R*-HETE is converted into lipoxins. The latter was the first type of lipid mediator known to start the resolution of inflammation. Thus, low-dose aspirin treatment will start resolution earlier than might be so otherwise (see chapter on ‘Specialised Pro-resolving Mediator Network’). Because most NSAIDs inhibit COXs reversibly, their use can delay complete resolution of inflammation.

### Resolvins

**Figure 12:**
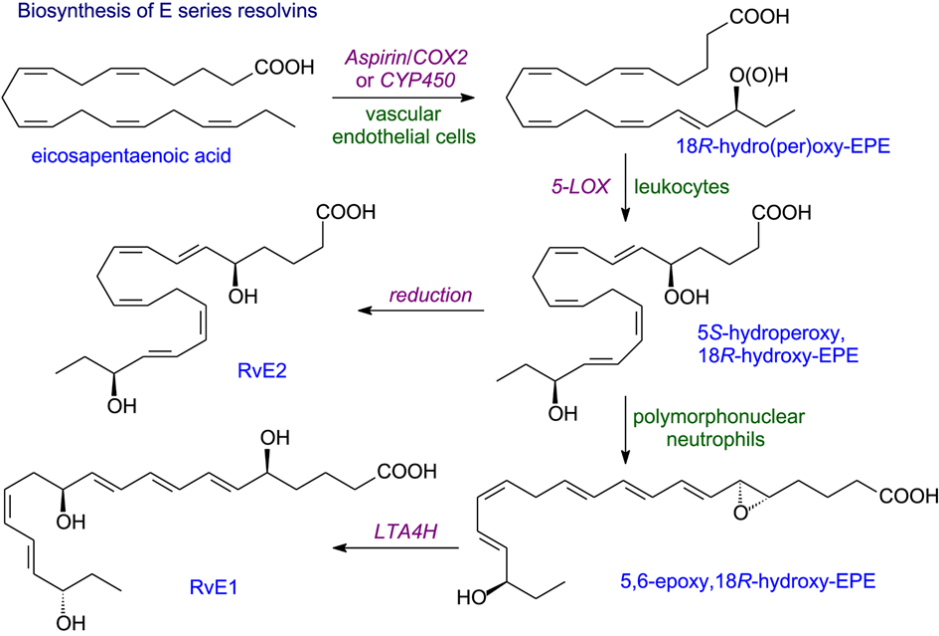
Biosynthesis of E-series resolvins

**Figure 13:**
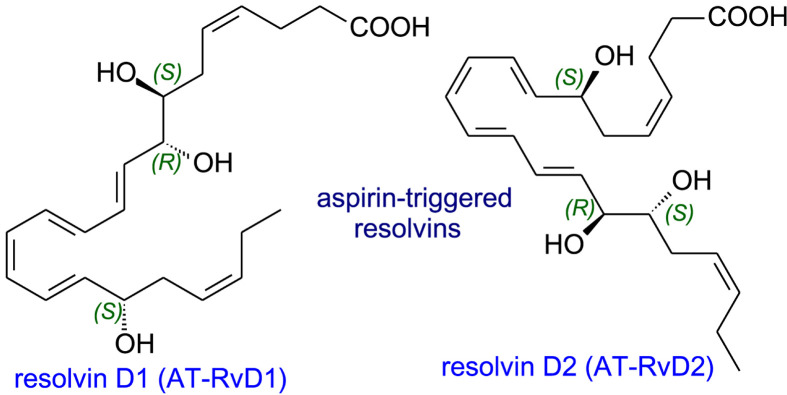
Aspirin-triggered resolvins

Resolvins (resolution phase interaction products) are produced from EPA, DPA and DHA and stop inflammation becoming chronic and, hence, prevent tissue damage and reduce the risk of a host of diseases arising [51,57]. COX-2 which has been acetylated by aspirin or a CYP 450 will introduce an 18*R*-hydroperoxy group into the EPA molecule (Figure 12). Reduction yields RvE2 while two steps are used for RvE1 formation. Since it is rare for all the enzymes needed to be found in a single cell, a trans-cellular sequence is needed as in the formation of lipoxins. Instead of EPA, DHA can be converted into 17*R*-resolvins by a similar aspirin-triggered COX-2 mechanism. The aspirin-triggered resolvins AT-RvD1 and AT-RvD2 (Figure 13) are formed when epoxidation takes place on 17*R*-hydroperoxy intermediate while a 5-LOX reaction with FLAP assistance forms AT-RvD3 or AT-RvD4.

Epimeric 18*S*-resolvins are also produced *in vivo* by related pathways with 15-LOX catalysing the first step (c.f. protectin formation) and these *S*-resolvins have their own distinct biological activities. Thus, in the absence of aspirin, 15-LOX generates 17*S*-hydroxyDHA which then goes on to form RvD1 and epimeric RvD2, both of which contain a 17*S*-hydroxyl group. A different intermediate from 17*S*-hydroxyDHA is transformed via an epoxide to RvD3 and RvD4. Both the products and intermediates from DHA have anti-inflammatory properties and two more resolvins, RvD5 and RvD6, have now been characterised.

In addition to EPA and DHA, DPA has been shown to be converted to three resolvins of which RvD1(*n*-3_DPA_) is the most abundant. Four further metabolites of DPA have a hydroxyl group at the 13-position and have been designated as 13-series resolvins (RvTs).

Tissue inactivation of resolvins has been studied best in the case of RvE1 and RVD1 where oxidation is key. For RvE1, at least four distinct oxidative pathways are involved*.*

### Maresins

**Figure 14:**
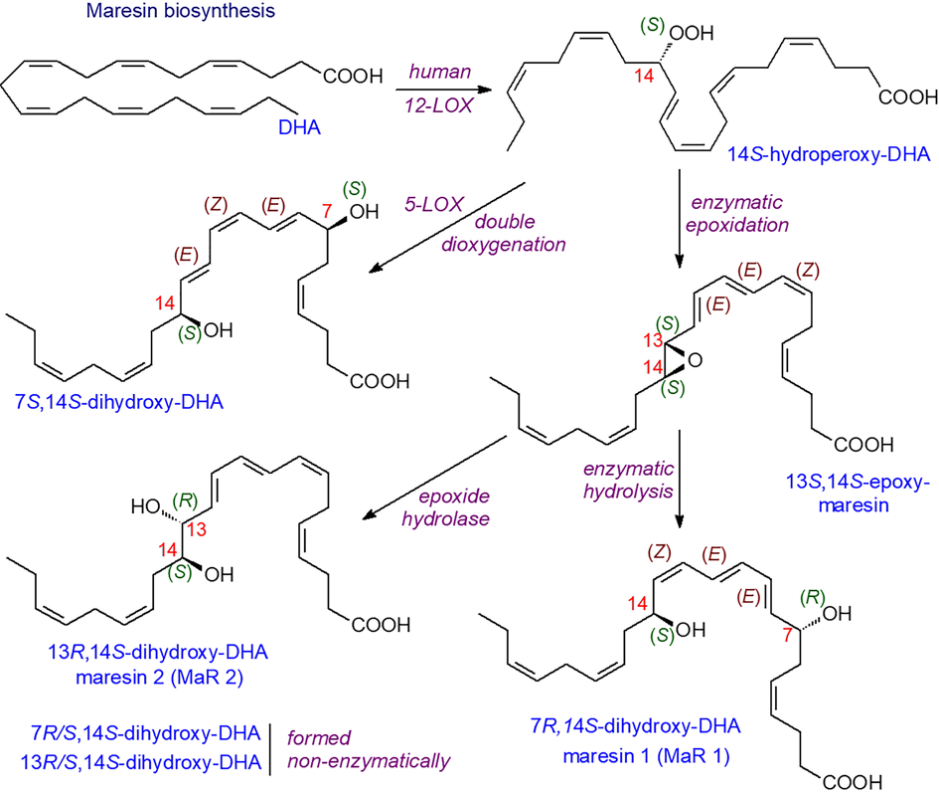
Biosynthesis of maresins

A single oxygenation by 12-LOX (in macrophages or platelets) to form a 14*S*-hydroperoxyDHA intermediate begins the formation of maresins (*ma*crophage mediator in *res*olving *in*flammation) as illustrated in Figure 14 [58,59]. Some details of the pathway are still ill-defined but both MaR1 and MaR2 have potent anti-inflammatory and pro-resolving activities. Similar oxygenated compounds with anti-inflammatory properties are formed from 22:5(*n*-3) and 22:5(*n*-6) fatty acids.

Some maresin-like di-oxygenated metabolites produced by sequential oxidation by 12-LOX and enzymes of the CYP450 family can occur in macrophages. For example, 14*S*, 21*S*-dihydroxy-docosa-4*Z*,7*Z*,10*Z*,12*E*,16*Z*,19*Z*-hexaenoic acid and its epimers can be produced. This compound is induced by wounding and has been shown to promote wound healing. Similar 14, 22-dihydroxy metabolites are synthesised in leukocytes and platelets and also promote wound healing.

Sulphido-peptide conjugated mediators (analogous to cysteinyl-leukotrienes) have been detected for the SPMs in macrophages, initially for maresins and then for protectins and resolvins [51]. The various compounds have been detected in lymph nodes, serum and milk in humans and shown to regulate bacterial clearance and the repair and regeneration of damaged tissues.

### Biological activities of SPMs

Given the diverse nature of SPMs, it is hardly surprising that they can give rise to a variety of biological effects. In general, their action is via specific G-protein coupled receptors which can lead to both rapid and long-term actions. Moreover, SPMs can antagonise pro-inflammatory receptors such as the leukotriene B4 receptor, BLT1.

As mentioned before, acute inflammation is a vital response to infection or tissue damage. However, the time scale needs to be kept as short as needed and chronic inflammation avoided. Indeed, occurrence of the latter will lead almost inevitably to tissue damage and loss of function. Under normal circumstances compounds involved in inflammation may initiate the resolution phase. For example, leukotriene LTB_4_ and prostaglandins, PGE_2_ and PGD_2_, will stimulate induction of 15-LOXs needed for later production of SPMs.

Early in inflammation there is a change in ARA metabolism from synthesis of leukotrienes to lipoxin formation. Local mobilisation of *n*-3 PUFAs, such as DHA, occurs followed by production of SPMs. As such mediators are formed, macrophages and mast cells remove excess neutrophils together with cell debris resulting from microorganisms and host defences. A noted ancillary effect is that low dose aspirin facilitates the resolution of inflammation by enhancing conversion of EPA and DHA to resolvins of the E and D series.

While the resolution process is on-going, a change in the phenotype of macrophages towards a pro-resolution state occurs. Initially, at the onset of inflammation, macrophages counter disease by removing invading pathogens by phagocytosis. However, these actions can cause trauma and tissue damage so SPMs, like lipoxins, have an important role in regulating and inhibiting these effects. Thus, RvE1 dramatically reduces dermal inflammation, peritonitis and interleukin production. A measure of its effectiveness is that it reduces inflammatory pain better than morphine! Furthermore, RvE2 has been shown to effectively reduce joint pain in arthritis. Similar beneficial effects of resolvins from DHA have been found (RvD2 ameliorating bacterial sepsis, RvD3 active in later stages of resolution, RvD4 helping clearance of apoptotic cells by skin fibroblasts).

The protectins have similar effects to resolvins but mainly in brain tissue [60–62]. They promote resolution of neuroinflammation and stimulate nerve regeneration. In animal models. PD1 has protective effects against stroke and Alzheimer’s disease. In non-neuronal tissues, PD1 promotes apoptosis of T cells, is beneficial towards asthma and may have potential in slowing viral replication. Protectins synthesised in white adipose tissue have anti-inflammatory effects on obesity and diabetes.

Maresin 1 is a powerful regulator of the resolution of inflammation, tissue regeneration and pain [62]. Notable effects are in lung, with vascular and metabolic diseases and bacterial infections. Maresins have been found especially important in tissue regeneration and wound healing in the latter stages of resolution. Indeed, they have been used successfully for specific surgical interventions.

Although SPMs are produced locally, they also reach the circulation and, therefore, may have effects in tissues other than where they are synthesised. Notably, SPMs have been found in bioactive concentrations in human milk and placenta. So, they may function in normal physiological development.

We have already highlighted the importance of SPMs in preventing chronic inflammatory diseases [63,64]. As a result of basic scientific knowledge, SPMs have been tested in a wide range of experimental models including peritonitis, colitis, arthritis, psoriasis, dry eye, cardiovascular disease, asthma and some cancers (see [65]). Although further clinical trials are necessary, it is hoped that the use of SPMs may be efficacious for many low-level inflammatory diseases. Particular attention is being paid to obesity [66], including insulin resistance, Type 2 diabetes, metabolic disease [67,68], non-alcoholic liver disease and cardiovascular complaints [69]. Overall, treatment of patients with a diet containing increased levels of very long chain *n*-3 PUFAs, together with low-dose aspirin may be a cost-effective method to ameliorate the clinical symptoms of many important disorders where inflammation is involved.

## Other oxidised lipids with biological activities

In recent years, it has become apparent that various other oxidised lipids can be formed by both enzymatic and non-enzymatic means. For example, isoprostanes, neuroprostanes and phytoprostanes [70] are covered in a later chapter. Enzymatically oxidised phospholipids can be produced by reactions in which eicosanoids are attached in immune cells. Such lipids are represented by various chemically distinct families with important bioactivities. Lipoxygenase activities are usually involved in the formation of these oxidised phospholipids [71–73] which have important regulatory roles in both health and disease. For example, they have been implicated in ferroptosis, apoptosis, blood clotting, arthritis, diabetes and cardiovascular disease [74].

## Production of VLCPUFAs from dietary essential fatty acids

The VLCPUFAs (>18C) which are almost the exclusive source of the lipid mediators discussed above are not produced in higher plants [75,76]. Instead, the vast majority are synthesised by marine microalgae at the base of the marine food web [77]. Nevertheless, even this supply is currently under threat due to climate change [4,78]. For humans, the precursor essential fatty acids (LA, ALA) can be converted to 20 and 22C PUFA but rather inefficiently [3,54]. Thus, a dietary supply of EPA and DHA is considered necessary (‘conditionally essential’) under certain circumstances [3] and, indeed, is considered desirable for all individuals, but especially for the newborn [79–82].

**Figure 15:**
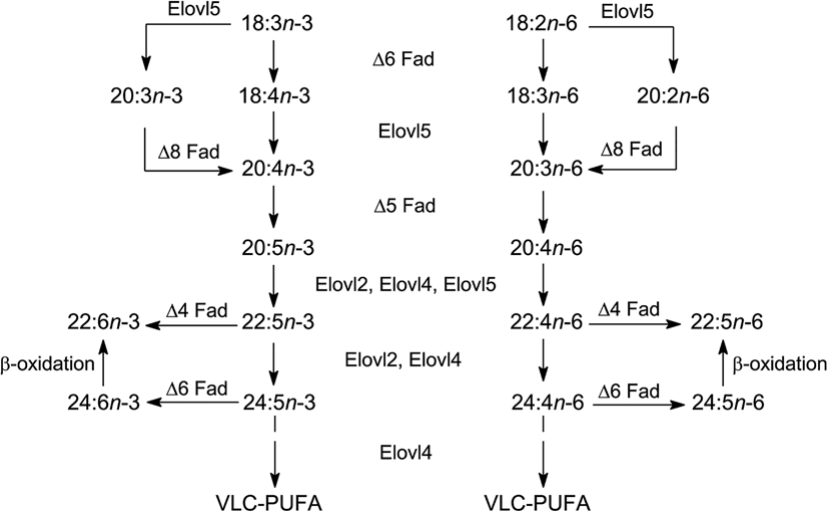
Conversion of the essential fatty acids to 20- or 22-carbon PUFAs (re-drawn from [90] with permission)

The essential fatty acids, LA and ALA are produced in photosynthetic organisms by specific desaturase enzymes (e.g. FAD3 and FAD7/8 for ALA). Then a series of desaturation and elongation reactions will convert LA to ARA or ALA to EPA and then DHA in both humans and fish (the usual source of VLCPUFA in our diets) (Figure 15). The most direct route to convert EPA to DHA is via an elongation to 22:5 and a Δ^4^-desaturase. However, Δ^4^-desaturase activity could not be detected in mammalian tissues and so the ‘Sprecher pathway’ involving a 24:6 intermediate followed by beta-oxidation was proposed (see [83]). A recent article has suggested a re-visiting of the situation [84] but, nevertheless, the Sprecher pathway seems to be key in humans and, with the exception of a few teleost species, in fish also [85]. Thus, because fish are the main dietary source of *n*-3 VLCPUFA for humans, it is important that they contain adequate levels of EPA and DHA, and oily fish sources should be from capture fisheries or from aquaculture with an adequate supply of *n*-3 VLCPUFA in the feed. Since aquaculture now supplies more than half the fish used [86] and some 75% of marine-sourced fish oils are currently used in aquaculture [87], it is easy to see that there is currently a serious problem that will only get worse as demand for EPA and DHA increases in the future [88].

For the conversion of LA and ALA into VLCPUFA, the same enzymes operate for both the *n*-3 and *n*-6 pathways (Figure 15). This means that the dietary ratio of *n*-6/*n*-3 PUFAs (mainly LA/ALA) in human diets is important. While many ‘Western diets’ have a ratio of over 10, for good health a ratio of 3-4 is recommended [4,54,77]. Furthermore, for some teleost fish the lack of a Δ^5^-desaturase means that conversion of ALA to EPA is not possible [89]. It should also be noted that expression of the necessary enzymes for VLCPUFA biosynthesis in fish is controlled by the diet [90].

In view of the problems faced in maintaining levels of EPA and DHA in fish being consumed by humans, alternative sources of these acids for aquaculture or, indeed, directly for human consumption have been sought. Thus, crops have been manipulated to allow the biosynthesis of EPA and/or DHA [88,91]. Two methods have been used. First, a polyketide synthase system was used to engineer oilseed rape to yield DHA [92]. This strategy mimicked the enzyme used by *Schizochytrium* to make the commercially successful oils used for infant formulations [93]. Second, various combinations of genes (mostly from microalgae) have been used to allow different higher plant systems to make EPA and/or DHA [94,95]. The success of these efforts in oilseed crops, especially oilseed rape (Canola) has been discussed [88]. Currently, there are a number of commercially available (or potentially available) new sources of EPA and DHA (see [88]).

As has been discussed before, adequate dietary n-3 VLCPUFAs has important implications for good health [96] and in the prevention of important complaints and illnesses [54,97–99]. These considerations will ensure that supply of the direct precursors (ARA, EPA, DHA) for lipid mediators will remain an important topic for years to come.

## Plant oxylipins

In plants LA and, especially, ALA give rise to bioactive molecules which are very important for functions such as in stress responses and development [100]. The ‘lipoxygenase pathway ‘is initiated by lipoxygenase (9-LOX or 13-LOX) attack on a PUFA which, in leaves, is mainly ALA. LOX enzymes in plants are stable proteins, often present in high amounts, especially in leaves.

The fatty acid hydroperoxides that are produced by plant LOXs can, themselves, be metabolised to yield three types of derivatives. By a co-oxidation with peroxidase they can form a mixture of epoxy and hydroxy fatty acids that have roles in cutin formation. In contrast, the hydroperoxides can be cleaved by hydroperoxide lyase to give an aldehyde and an oxo-unsaturated fatty acid. The emission of C_6_ aldehydes and alcohols occurs rapidly in plants in response to wounding, and they contribute to protection against the invasion of fungi and insects, while taking part in abiotic stress responses by inducing the expression of stress-associated genes.The third reaction with hydroperoxides is by allene oxide synthase. This forms 12-oxophytodienoic acid which is the precursor for jasmonic acid and other jasmonates (with intriguing structural similarities to the cyclopentenone prostaglandins). These important effectors of growth, development and senescence are described in detail in a later chapter (‘Jasmonates’). Some recent updates on jasmonate functions can be found in a special edition of Plant and Cell Physiology [101–106].

## Fatty acid esters of hydroxy fatty acids (FAHFA)

A range of unesterified fatty acids containing a hydroxyl group to which a further fatty acid is esterified have been found in the adipose tissue, serum, milk and many other tissues of mice and humans. These include several regio-isomers of the hydroxyl component as well as many different ester-linked fatty acids [107]. While little is yet known of the biosynthesis of these lipids, it has been established that they are produced endogenously with defined stereochemistry, i.e. the hydroxyl group has the *R*-configuration and peroxisomal enzymes are believed to be involved. Adipose tissue triacylglycerols act as a reservoir for these compounds, which have been classed as lipokines, i.e. lipid molecules derived from adipose tissue that act as hormonal regulators and coordinate an array of cellular processes.

Among those biological activities described to date, FAHFA have anti-diabetic and anti-inflammatory effects, even when administered orally and they protect against colitis by regulating gut innate and adaptive immune responses. They are believed to have an important role in maintaining normal blood sugar levels and insulin sensitivity, possibly by acting as selective agonists for the GPR40 and GPR120 receptors [108].

FAHFA have recently been reported in plants also [108] and have been comprehensively reviewed recently [109].

## Summary

- Oxylipins, formed from *n*-3 and *n*-6 polyunsaturated fatty acids, are important lipid mediators.
- Three types of oxidase (cyclooxygenases, lipoxygenases, cytochrome P450 oxidases) are involved in their formation.
- Oxylipins bind to receptors, are rapidly catabolised and usually act where they are produced—-as ‘local’ hormones.
- Oxylipins have multiple functions in mammalian health and disease, as well as in other animals and plants.

## Open Access

Open access for this article was enabled by the participation of Cardiff University in an all-inclusive *Read & Publish* pilot with Portland Press and the Biochemical Society under a transformative agreement with JISC.

## Abbreviations

ALA: α-linolenic
ARA: arachidonic
DHA: docosahexaenoic
EFA: essential fatty acid
EPA: eicosapentaenoic
FAHFA: fatty acid esters of hydroxy fatty acids
LA: linoleic
PUFA: polyunsaturated fatty acid
SPM: specialised pro-resolving mediator
